# Supplementary material for: Infant Gut Microbiota Associated with Fine Motor Skills
Source: Nutrients. 2021 May 14;13(5):1673. doi: 10.3390/nu13051673 (PMC8156744; doi:10.3390/nu13051673)
Supplement: Supplementary file 1 [file nutrients-13-01673-s001.zip › Supplementary Material/Supplementary Tables Fine Motricity Nutrients_030521.docx]

**Table S1. General characteristics of the studied population**

| **Maternal characteristics** |  |
| --- | --- |
| **Age (years)** | 33.3 (4.17) |
| **Pre-pregnancy BMI** |  |
| Normal-Weight | 21 (28.58%) |
| Overweight | 33 (46.48%) |
| Obesity | 17 (23.94%) |
| **Pre-pregnancy weight (kg)** | 72.27 (14.7) |
| **Weight gain (kg)** | 8.51 (6.33) |
| **Diabetes** |  |
| Yes | 29 (40.85%) |
| No | 42 (59.15%) |
| **Education** |  |
| Primary/Secondary | 38 (55.07%) |
| University | 31 (44.93%) |
| **Smoking during pregnancy** |  |
| Yes | 6 (8.45%) |
| No | 65 (91.55%) |
| **Drinking alcohol during pregnancy** |  |
| Yes | 2 (2.82%) |
| No | 69 (97.18%) |
| **Type of delivery** |  |
| Eutocic | 42 (59.15%) |
| Dystocic | 9 (12.68%) |
| Cesarean | 20 (28.17%) |
| **Infant characteristics** |  |
| **Sex** |  |
| Male | 45 (63.38%) |
| Female | 26 (36.62%) |
| **Birth weight (g)** | 3272.68 (529.72) |
| **Birth length (cm)** | 50.2 (2.02) |
| **Birth head Circumference (cm)** | 34.69 (1.39) |
| **Placenta (g)** | 517.21 (119.79) |
| **Breastfeeding** |  |
| Exclusive | 43 (60.56%) |
| Mixed | 9 (12.68%) |
| Artificial | 19 (26.76%) |

Values listed are total for the variable (percent of total value n) or Means (SD).

**Table S2. Distribution of Bayley®-III scores of infant development in 71 full-term healthy infants at the age of 18 months**

| Receptive language | n | Expressive language | n | Fine motor | n | Gross motor | n | Composite cognitive | n | Composite language | n |
| --- | --- | --- | --- | --- | --- | --- | --- | --- | --- | --- | --- |
| 8 | 1 | 6 | 3 | 8 | 2 | 7 | 1 | 90 | 1 | 83 | 1 |
| 9 | 1 | 7 | 3 | 9 | 2 | 8 | 2 | 95 | 1 | 89 | 1 |
| 10 | 7 | 8 | 6 | 10 | 4 | 10 | 9 | 100 | 1 | 91 | 1 |
| 11 | 19 | 9 | 7 | 11 | 7 | 11 | 19 | 105 | 6 | 94 | 5 |
| **12*** | 25 | 10 | 13 | 12 | 16 | **12*** | 14 | 110 | 2 | 97 | 5 |
| 13 | 12 | **11*** | 22 | **13*** | 14 | 13 | 14 | 115 | 9 | 100 | 2 |
| 14 | 5 | 12 | 7 | 14 | 17 | 14 | 1 | 120 | 8 | 103 | 14 |
| 15 | 1 | 13 | 6 | 15 | 6 | 15 | 9 | **125*** | 15 | **106*** | 9 |
|  |  | 14 | 3 | 16 | 1 | 16 | 1 | 130 | 11 | 109 | 13 |
|  |  | 15 | 1 | 17 | 1 | 17 | 1 | 135 | 12 | 112 | 8 |
|  |  |  |  | 19 | 1 |  |  | 140 | 5 | 115 | 4 |
|  |  |  |  |  |  |  |  |  |  | 118 | 3 |
|  |  |  |  |  |  |  |  |  |  | 121 | 4 |
|  |  |  |  |  |  |  |  |  |  | 124 | 1 |

* Median scores for each Bayley®-III scale

**Table S4. Association between a-diversity indexes and each Bayley®-III scale. T-Student test.**

|  |  | Observed OTUs | | PD Whole Tree | | SDI | |
| --- | --- | --- | --- | --- | --- | --- | --- |
|  |  | Below the median | Above the median | Below the median | Above the median | Below the median | Above the median |
| Composite language | Mean  (SD) | 247.07  (62.39) | 259  (54.35) | 14.22  (2.42) | 14.43  (2.18) | 2.87  (0.74) | 3.04  (0.52) |
|  | *p*-value | 0.394 | | 0.705 | | 0.245 | |
| Expressive language | Mean  (SD) | 244.67  (51.67) | 262.03  (62.11) | 14.28  (2.07) | 14.4  (2.46) | 2.88  (0.68) | 3.05  (0.56) |
|  | *p*-value | 0.209 | | 0.814 | | 0.242 | |
| Receptive language | Mean  (SD) | 256.14  (61.27) | 252.45  (55.9) | 14.71  (2.41) | 14.1  (2.16) | 2.93  (0.51) | 3  (0.7) |
|  | *p*-value | 0.794 | | 0.268 | | 0.681 | |
| Fine motor | Mean  (SD) | 253.32  (56.74) | 254.45  (59.25) | 14.42  (2.31) | 14.28  (2.27) | 2.95  (0.48) | 2.98  (0.72) |
|  | *p*-value | 0.936 | | 0.802 | | 0.828 | |
| Gross motor | Mean  (SD) | 254.77  (60.26) | 253.33  (56.51) | 14.25  (2.61) | 14.42  (2) | 2.97  (0.66) | 2.97  (0.6) |
|  | *p*-value | 0.917 | | 0.758 | | 0.965 | |
| Composite cognitive | Mean  (SD) | 253.5  (75.17) | 254.26  (43.89) | 14.39  (2.84) | 14.32  (1.84) | 2.97  (0.74) | 2.97  (0.54) |
|  | *p*-value | 0.957 | | 0.898 | | 0.978 | |

PD Whole Tree: Faith’s Phylogenetic Diversity Whole Tree; SDI: Shannon Diversity Index

**Table S5. Associations between gut microbiota composition (16S rDNA gene sequences) and Bayley®-III scales according to the weighted UniFrac.**

|  | F model | R^2^ | *p* value |
| --- | --- | --- | --- |
| Composite language | 0.546 | 0.008 | 0.816 |
| Expressive language | 0.487 | 0.007 | 0.846 |
| Receptive language | 0.758 | 0.011 | 0.517 |
| Fine motor | 2.877 | 0.04 | **0.021*** |
| Gross motor | 0.632 | 0.009 | 0.699 |
| Composite cognitive | 0.892 | 0.013 | 0.469 |

**p*-values ≤ 0.05 are highlighted in bold

**Table S6. Associations between gut microbiota composition (16S rDNA gene sequences) and Bayley®-III scales according to the unweighted UniFrac.**

|  | F model | R^2^ | *p* value |
| --- | --- | --- | --- |
| Composite language | 0.785 | 0.011 | 0.895 |
| Expressive language | 0.992 | 0.014 | 0.481 |
| Receptive language | 0.744 | 0.011 | 0.944 |
| Fine motor | 0.956 | 0.014 | 0.552 |
| Gross motor | 0.876 | 0.013 | 0.743 |
| Composite cognitive | 0.985 | 0.014 | 0.502 |

**Table S7. Associations between gut microbiota composition (16S rDNA gene sequences) and other variables of interest according to the weighted UniFrac.**

|  | F model | R^2^ | *p* value |
| --- | --- | --- | --- |
| Maternal age | 0.609 | 0.008 | 0.720 |
| Pre-pregnancy BMI | 4.434 | 0.059 | **0.005*** |
| Pre-pregnancy weight | 0.550 | 0.007 | 0.770 |
| Maternal diabetes | 1.581 | 0.021 | 0.126 |
| Smoking during pregnancy | 0.248 | 0.003 | 0.989 |
| Drinking alcohol during pregnancy | 1.012 | 0.013 | 0.393 |
| Type of delivery | 1.019 | 0.027 | 0.405 |
| Infant sex | 0.880 | 0.012 | 0.446 |
| Birth weight | 1.247 | 0.016 | 0.242 |
| Breastfeeding up to 3 months | 2.862 | 0.038 | **0.023*** |
| Breastfeeding up to 6 months | 0.766 | 0.010 | 0.569 |

**p*-values ≤ 0.05 are highlighted in bold
