# Supplementary material for: Infant Gut Microbiota Associated with Fine Motor Skills
Source: Nutrients. 2021 May 14;13(5):1673. doi: 10.3390/nu13051673 (PMC8156744; doi:10.3390/nu13051673)
Supplement: Supplementary file 1 [file nutrients-13-01673-s001.zip › Supplementary Tables Fine Motricity Nutrients_030521.pdf]

Table S1. General characteristics of the studied population

**Maternal characteristics**	
**Age (years)**	33.3 (4.17)
**Pre-pregnancy BMI**	
Normal-Weight	21 (28.58%)
Overweight	33 (46.48%)
Obesity	17 (23.94%)
**Pre-pregnancy weight (kg)**	72.27 (14.7)
**Weight gain (kg)**	8.51 (6.33)
**Diabetes**	
Yes	29 (40.85%)
No	42 (59.15%)
**Education**	
Primary/Secondary	38 (55.07%)
University	31 (44.93%)
**Smoking during pregnancy**	
Yes	6 (8.45%)
No	65 (91.55%)
**Drinking alcohol during pregnancy**	
Yes	2 (2.82%)
No	69 (97.18%)
**Type of delivery**	
Eutocic	42 (59.15%)
Dystocic	9 (12.68%)
Cesarean	20 (28.17%)
**Infant characteristics**	
**Sex**	
Male	45 (63.38%)
Female	26 (36.62%)
**Birth weight (g)**	3272.68 (529.72)
**Birth length (cm)**	50.2 (2.02)
**Birth head Circumference (cm)**	34.69 (1.39)
**Placenta (g)**	517.21 (119.79)
**Breastfeeding**	
Exclusive	43 (60.56%)
Mixed	9 (12.68%)
Artificial	19 (26.76%)

Values listed are total for the variable (percent of total value n) or Means (SD).

Table S2. Distribution of Bayley®-III scores of infant development in 71 full-term healthy infants at the age of 18 months

Receptive language	n	Expressive language	n	Fine motor	n	Gross motor	n	Composite cognitive	n	Composite language	n
8	1	6	3	8	2	7	1	90	1	83	1
9	1	7	3	9	2	8	2	95	1	89	1
10	7	8	6	10	4	10	9	100	1	91	1
11	19	9	7	11	7	11	19	105	6	94	5
**12***	25	10	13	12	16	**12***	14	110	2	97	5
13	12	**11***	22	**13***	14	13	14	115	9	100	2
14	5	12	7	14	17	14	1	120	8	103	14
15	1	13	6	15	6	15	9	**125***	15	**106***	9
		14	3	16	1	16	1	130	11	109	13
		15	1	17	1	17	1	135	12	112	8
				19	1			140	5	115	4
										118	3
										121	4
										124	1

* Median scores for each Bayley®-III scale

Table S4. Association between a-diversity indexes and each Bayley®-III scale. T-Student test.

		Observed OTUs		PD Whole Tree		SDI	
		Below the median	Above the median	Below the median	Above the median	Below the median	Above the median
Composite language	Mean (SD)	247.07 (62.39)	259 (54.35)	14.22 (2.42)	14.43 (2.18)	2.87 (0.74)	3.04 (0.52)
	p-value	0.394		0.705		0.245	
Expressive language	Mean (SD)	244.67 (51.67)	262.03 (62.11)	14.28 (2.07)	14.4 (2.46)	2.88 (0.68)	3.05 (0.56)
	p-value	0.209		0.814		0.242	
Receptive language	Mean (SD)	256.14 (61.27)	252.45 (55.9)	14.71 (2.41)	14.1 (2.16)	2.93 (0.51)	3 (0.7)
	p-value	0.794		0.268		0.681	
Fine motor	Mean (SD)	253.32 (56.74)	254.45 (59.25)	14.42 (2.31)	14.28 (2.27)	2.95 (0.48)	2.98 (0.72)
	p-value	0.936		0.802		0.828	
Gross motor	Mean (SD)	254.77 (60.26)	253.33 (56.51)	14.25 (2.61)	14.42 (2)	2.97 (0.66)	2.97 (0.6)
	p-value	0.917		0.758		0.965	
Composite cognitive	Mean (SD)	253.5 (75.17)	254.26 (43.89)	14.39 (2.84)	14.32 (1.84)	2.97 (0.74)	2.97 (0.54)
	p-value	0.957		0.898		0.978	

PD Whole Tree: Faith's Phylogenetic Diversity Whole Tree; SDI: Shannon Diversity Index

Table S5. Associations between gut microbiota composition (16S rDNA gene sequences) and Bayley®-III scales according to the weighted UniFrac.

	F model	R^2	*p* value
Composite language	0.546	0.008	0.816
Expressive language	0.487	0.007	0.846
Receptive language	0.758	0.011	0.517
Fine motor	2.877	0.04	**0.021***
Gross motor	0.632	0.009	0.699
Composite cognitive	0.892	0.013	0.469

**p*-values ≤ 0.05 are highlighted in bold

Table S6. Associations between gut microbiota composition (16S rDNA gene sequences) and Bayley®-III scales according to the unweighted UniFrac.

	F model	R^2	*p* value
Composite language	0.785	0.011	0.895
Expressive language	0.992	0.014	0.481
Receptive language	0.744	0.011	0.944
Fine motor	0.956	0.014	0.552
Gross motor	0.876	0.013	0.743
Composite cognitive	0.985	0.014	0.502

Table S7. Associations between gut microbiota composition (16S rDNA gene sequences) and other variables of interest according to the weighted UniFrac.

	F model	R^2	*p* value
Maternal age	0.609	0.008	0.720
Pre-pregnancy BMI	4.434	0.059	**0.005***
Pre-pregnancy weight	0.550	0.007	0.770
Maternal diabetes	1.581	0.021	0.126
Smoking during pregnancy	0.248	0.003	0.989
Drinking alcohol during pregnancy	1.012	0.013	0.393
Type of delivery	1.019	0.027	0.405
Infant sex	0.880	0.012	0.446
Birth weight	1.247	0.016	0.242
Breastfeeding up to 3 months	2.862	0.038	**0.023***
Breastfeeding up to 6 months	0.766	0.010	0.569

**p*-values ≤ 0.05 are highlighted in bold
